# Abdominal Wall Abscess Due to Retained Gallstone After Laparoscopic Cholecystectomy: A Case Report

**DOI:** 10.7759/cureus.115670

**Published:** 2026-09-02

**Authors:** Nafer Anaya, Sebastian Preciado, Camilo Garcia, Julian Arenas Trujillo, Laura Sanchez

**Affiliations:** 1 Surgery, Clínica La Colina, Bogotá, COL; 2 Epidemiology, Clínica La Colina, Bogotá, COL; 3 Radiology, Universidad Nacional de Colombia, Bogotá, COL

**Keywords:** gallstone migration, gallstones, postoperative complications, spilled gallstones, subphrenic abscess

## Abstract

Laparoscopic cholecystectomy (LC) is the gold standard treatment for cholelithiasis and cholecystitis; however, gallbladder perforation with spillage of gallstones may occur and result in delayed complications. We present the case of a 47-year-old woman who developed a chronic right abdominal wall mass and pain eight years after LC. Imaging revealed a right subdiaphragmatic collection extending into the abdominal wall, initially raising concern for a neoplastic process. Biopsies were inconclusive, and due to persistent symptoms, laparoscopic exploration was performed, revealing severe adhesions and a subdiaphragmatic abscess extending into the abdominal wall, with a retained gallstone, which was successfully removed. The patient had an uneventful postoperative course, with progressive resolution of the inflammatory changes and abdominal wall mass. This case highlights the potential for retained gallstones to cause delayed inflammatory complications and mass-like lesions years after LC, potentially mimicking neoplastic disease. A history of previous LC should therefore be considered when evaluating unexplained chronic abdominal or abdominal wall collections.

## Introduction

Laparoscopic cholecystectomy (LC) has become the gold standard for the management of cholelithiasis and cholecystitis. Compared with open surgery, LC offers several well-established benefits, including reduced postoperative pain, shorter hospital stays, and a lower risk of surgical site infection. However, the use of smaller access ports may increase the risk of gallbladder perforation during extraction. Gallbladder perforation has been reported in approximately 36% of laparoscopic cholecystectomies [1], although its true incidence may be underestimated because gallbladder perforation and stone spillage are not consistently reported.

Gallbladder perforation may result in the spillage and subsequent retention of gallstones within the peritoneal cavity. This complication is particularly relevant in patients with gangrenous cholecystitis or gallbladder empyema, in whom infected or contaminated stones may be present. Although most spilled gallstones remain clinically silent, retained stones can cause delayed complications, including intra-abdominal abscesses, fistulas, and other inflammatory processes. The reported incidence of clinically significant complications varies considerably among studies, ranging from 0.08% to 0.3% in earlier series [2] and from 0.04% to 19% in more recent reports [3]. This wide variation likely reflects differences in study design, reporting practices, follow-up duration, and the definitions used for gallstone spillage and clinically significant complications.

A systematic review by Gavriilidis et al. identified 75 studies describing complications associated with spilled gallstones, with clinical manifestations occurring from as early as 2 days to as late as 15 years after LC [4]. Approximately 10% of patients were asymptomatic, whereas 90% developed symptomatic complications. Surgical intervention was required in 87% of symptomatic cases, while percutaneous drainage guided by ultrasound or computed tomography was performed in 12% [4].

Abscess formation is the most frequently reported complication, accounting for 36.5% of cases of intra-abdominal abscesses, followed by abdominal wall abscesses (10.9%) and retroperitoneal abscesses (9.4%). Collectively, abscesses accounted for approximately 56.5% of reported complications (4). Other less common manifestations, including thoracic empyema, have also been described [5].

In the Colombian literature, complications associated with retained gallstones have been reported since 2003, including peritoneocutaneous fistulas and retroperitoneal abscesses [6]. More recently, incidental retained gallstones discovered during subsequent surgical procedures, such as inguinal hernia repair, have also been reported [7].

Here, we present a case of a delayed complication associated with a retained gallstone following LC, highlighting the potential for spilled gallstones to cause clinically significant complications years after the initial procedure.

## Case presentation

A 47-year-old woman with a history of LC performed in 2017 for acute cholecystitis presented with progressive right subcostal pain and a gradually enlarging mass. The original operative report from the 2017 cholecystectomy was not available for review. Her symptoms began in October 2024, with localized right subcostal pain without other associated symptoms. A soft-tissue ultrasound performed at an outside institution at that time showed no significant abnormalities, and she was discharged. In February 2025, she noted the development of a palpable mass at the site of the pain, without fever. In June 2025, an outpatient abdominal magnetic resonance imaging (MRI) examination was performed using a 1.5-T Philips system (Philips Healthcare, Best, the Netherlands) and revealed a 110-cm³ heterogeneous, thin-walled, septated subdiaphragmatic collection anterior to the right hepatic lobe, with peripheral contrast enhancement and diffusion restriction, producing mass effect on the liver. A small 8 × 5 mm low-signal-intensity nodular structure on T2-weighted images was also identified within the collection, raising suspicion for a possible foreign body (Figures 1A-1H). 

**Figure 1:**
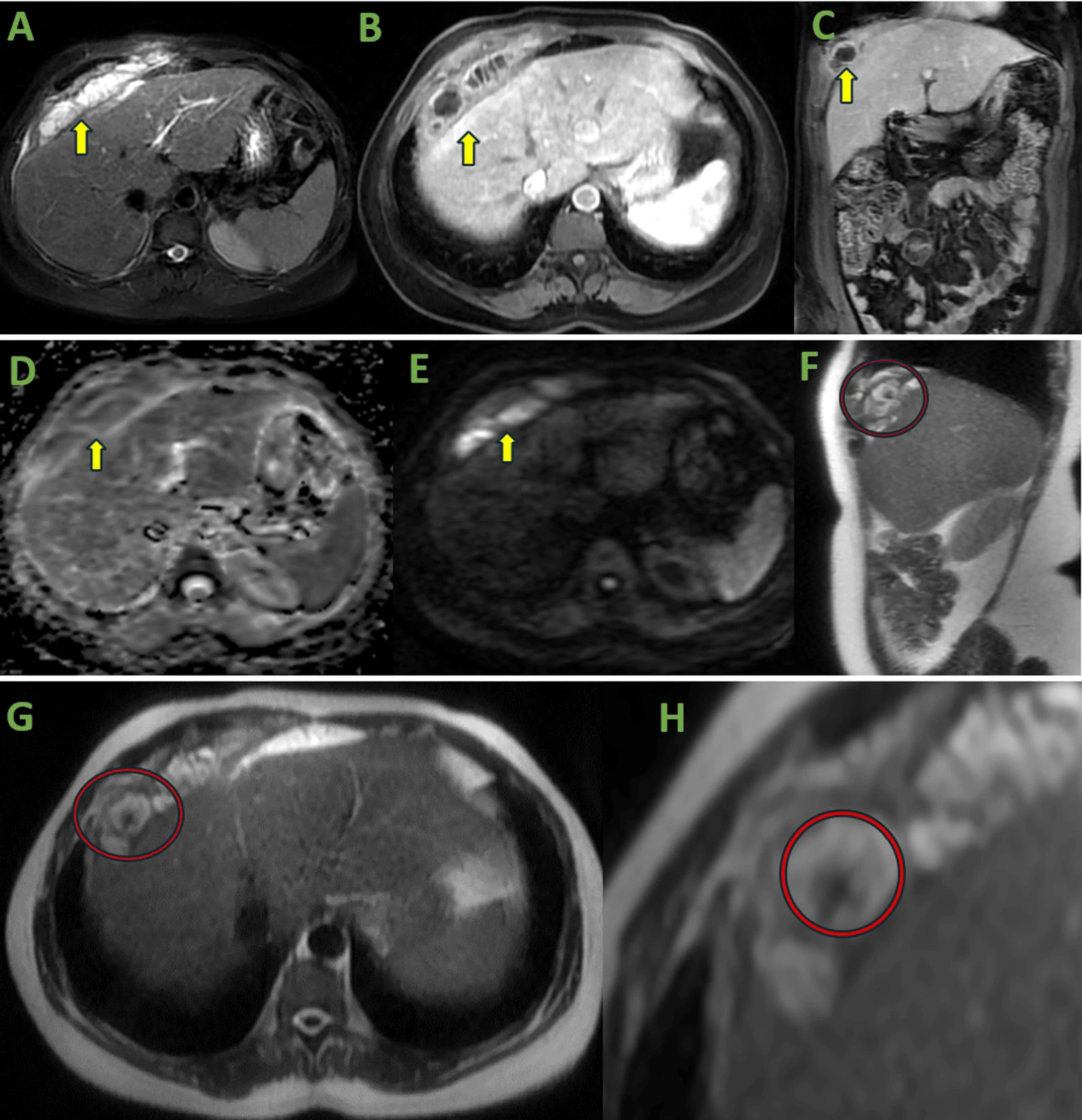
Contrast-enhanced abdominal magnetic resonance imaging (A) Axial T2-weighted image showing a subdiaphragmatic collection anterior to the right hepatic lobe, with thickened walls and internal septations. (B and C) Axial and coronal contrast-enhanced images in the portal venous phase demonstrating enhancement of the walls and septa. (D and E) Axial apparent diffusion coefficient and diffusion-weighted images showing restricted diffusion within the collection. (F, G, and H) Sagittal and axial images demonstrating the aforementioned collection with an 8 × 5 mm low-signal-intensity nodular structure on T2-weighted imaging, corresponding to a possible foreign body (blue and red circles).

Fine-needle aspiration and percutaneous drainage were subsequently performed. Histopathology showed severe acute inflammation without evidence of malignancy. Culture grew *Escherichia coli* with an AmpC β-lactamase resistance pattern, susceptible to cefepime and carbapenems. Despite these findings, the patient reported not receiving antibiotic therapy at that time.

In July 2025, because of persistent pain and progressive enlargement of the mass, a repeat soft-tissue ultrasound demonstrated a 5.9-cm³ heterogeneous collection located 10 mm from the skin surface, with preserved muscular planes (this measurement represented only the soft-tissue component accessible to ultrasound and did not include the deeper subdiaphragmatic component previously demonstrated on MRI). She was subsequently referred to the emergency department of our institution.

On admission, the patient reported worsening right subcostal pain limiting physical activity and right lateral decubitus, progressive enlargement of the mass, local erythema, and intermittent unmeasured fever for three weeks. She denied constitutional symptoms, including weight loss, night sweats, or other B symptoms. Her family history was notable for her father with gastric cancer, her mother with splenic cancer, a sister who died of colon cancer at 40 years of age, and a grandfather with lung cancer. She denied alcohol consumption, smoking, or recreational drug use.

She was tachycardic but afebrile and normotensive. Physical examination revealed an approximately 8-cm indurated right subcostal mass with overlying erythema and mild tenderness, without purulent drainage or signs of peritoneal irritation. In light of the progressive inflammatory changes and the previous isolation of *E. coli* with an AmpC β-lactamase resistance pattern, empirical antibiotic therapy with ertapenem was initiated on admission.

Given the gastrointestinal origin of the previously isolated organism and the progressive enlargement of the inflammatory mass with extension from the subdiaphragmatic region into the abdominal wall, a fistulous process connecting the collection to the gastrointestinal tract was considered. Contrast-enhanced computed tomography was performed using an 80-slice Revolution EVO system (GE HealthCare, Chicago, IL, USA) and demonstrated a 126-cm³ right subdiaphragmatic collection extending into the right abdominal wall (Figures 2A-2C).

**Figure 2:**
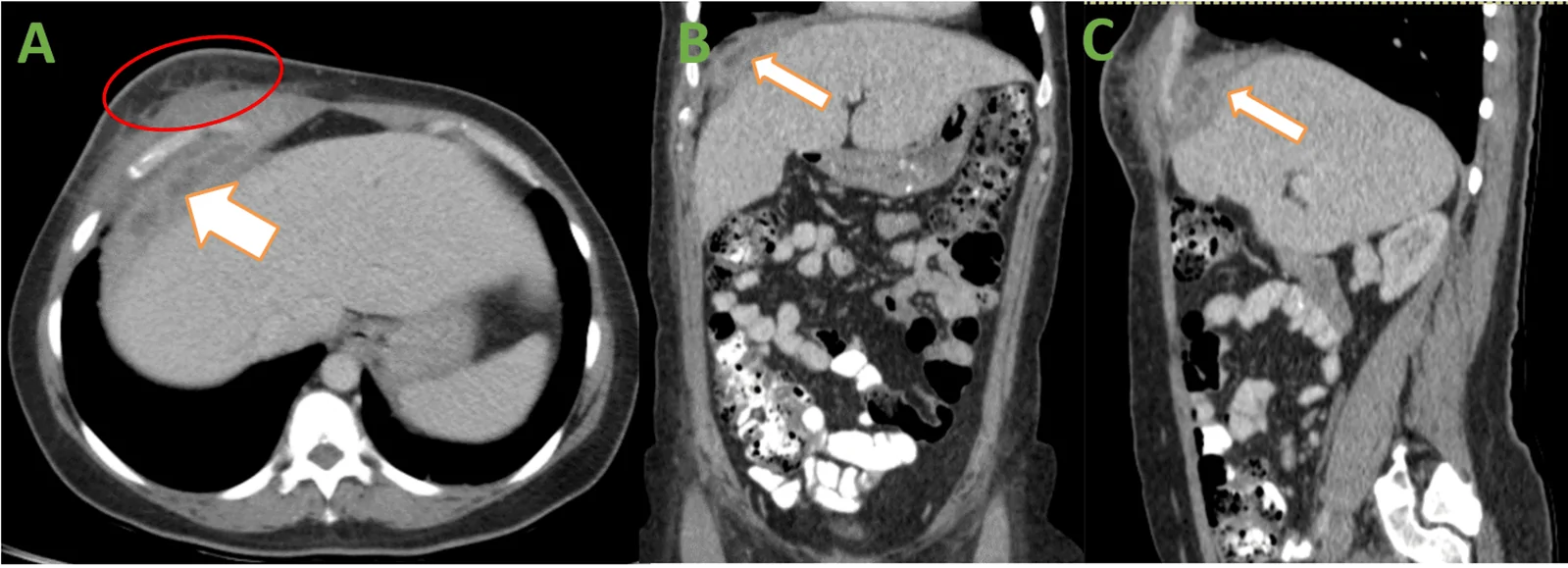
Contrast-enhanced computed tomography (CT) of the abdomen in soft-tissue window (A) Axial, (B) coronal, and (C) sagittal images demonstrating a heterogeneous right subdiaphragmatic collection extending into the abdominal wall, measuring 126 cm³ (white arrows). In (A), increased thickness and attenuation of the soft tissues of the abdominal wall are also demonstrated (red ellipse).

Because of the infiltrative appearance of the lesion and the patient's significant family history of malignancy, a neoplastic process involving the abdominal wall and adjacent costal structures was initially considered, including a primary soft-tissue or osseous neoplasm. The case was discussed in a multidisciplinary meeting with thoracic surgery and breast and soft-tissue surgery, and ultrasound-guided core-needle biopsy and percutaneous drainage were recommended. Cultures were negative for aerobic and anaerobic bacteria, fungi, and mycobacteria.

Given the chronic inflammatory course, repeated biopsies showing no malignancy, partial response to empirical antibiotic therapy, subsequent negative cultures obtained after antibiotic exposure, and the history of LC, a chronic abdominal abscess secondary to a retained gallstone was considered. The patient subsequently underwent diagnostic laparoscopy. Intraoperatively, severe adhesions involving the hepatic flexure of the colon, liver, and parietal peritoneum adjacent to the right costal margin were identified. The collection was drained, and a yellowish gallstone measuring approximately 1 cm was retrieved from the inflammatory cavity and removed in a sterile retrieval bag (Figures 3A-3C). Intraoperative cultures for aerobic and anaerobic bacteria were negative; these samples were obtained after the patient had received empirical antibiotic therapy. Mycobacterial polymerase chain reaction was also negative, as were Ziehl-Neelsen, periodic acid-Schiff, and Giemsa stains.

**Figure 3:**
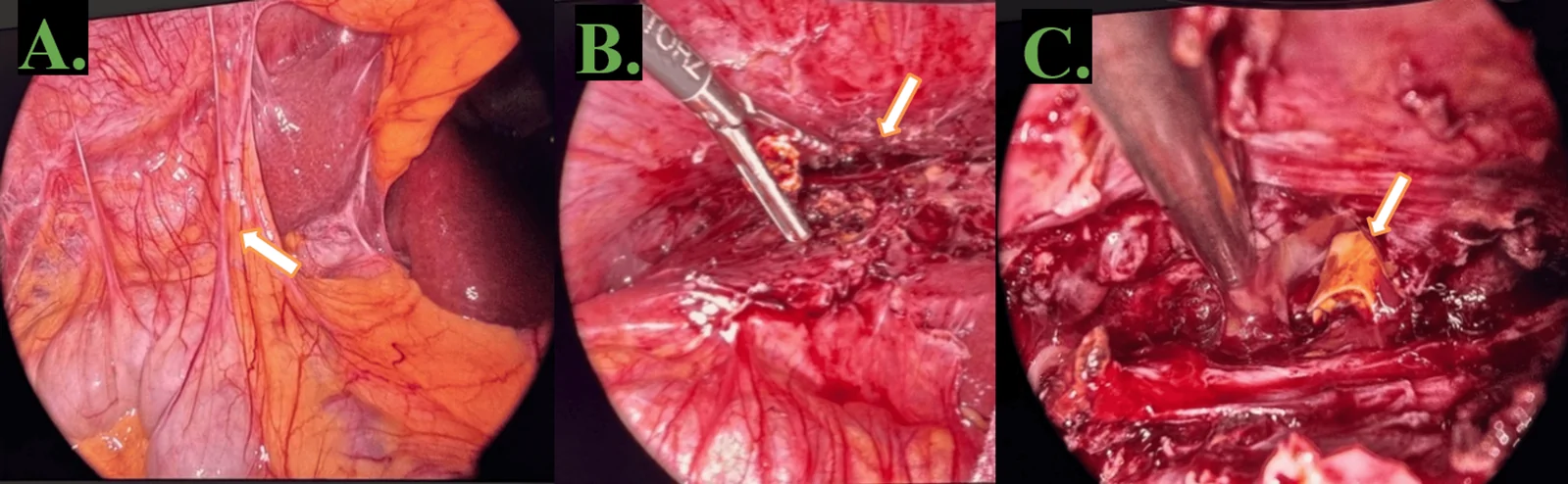
Intraoperative findings (A) Severe adhesions (white arrows) involving the free edge of the liver, colon, and parietal peritoneum. (B) The residual abscess cavity (white arrow) is observed above the liver and extending toward the right costal margin. (C) Close-up view of the retained gallstone (white arrow) within the abscess cavity.

The patient had an uneventful postoperative course and completed antibiotic therapy with ertapenem before discharge. At one-month follow-up, there was marked improvement in the right subcostal inflammation and induration, with well-healed surgical wounds and no evidence of recurrent infection (Figures 4A, 4B).

**Figure 4:**
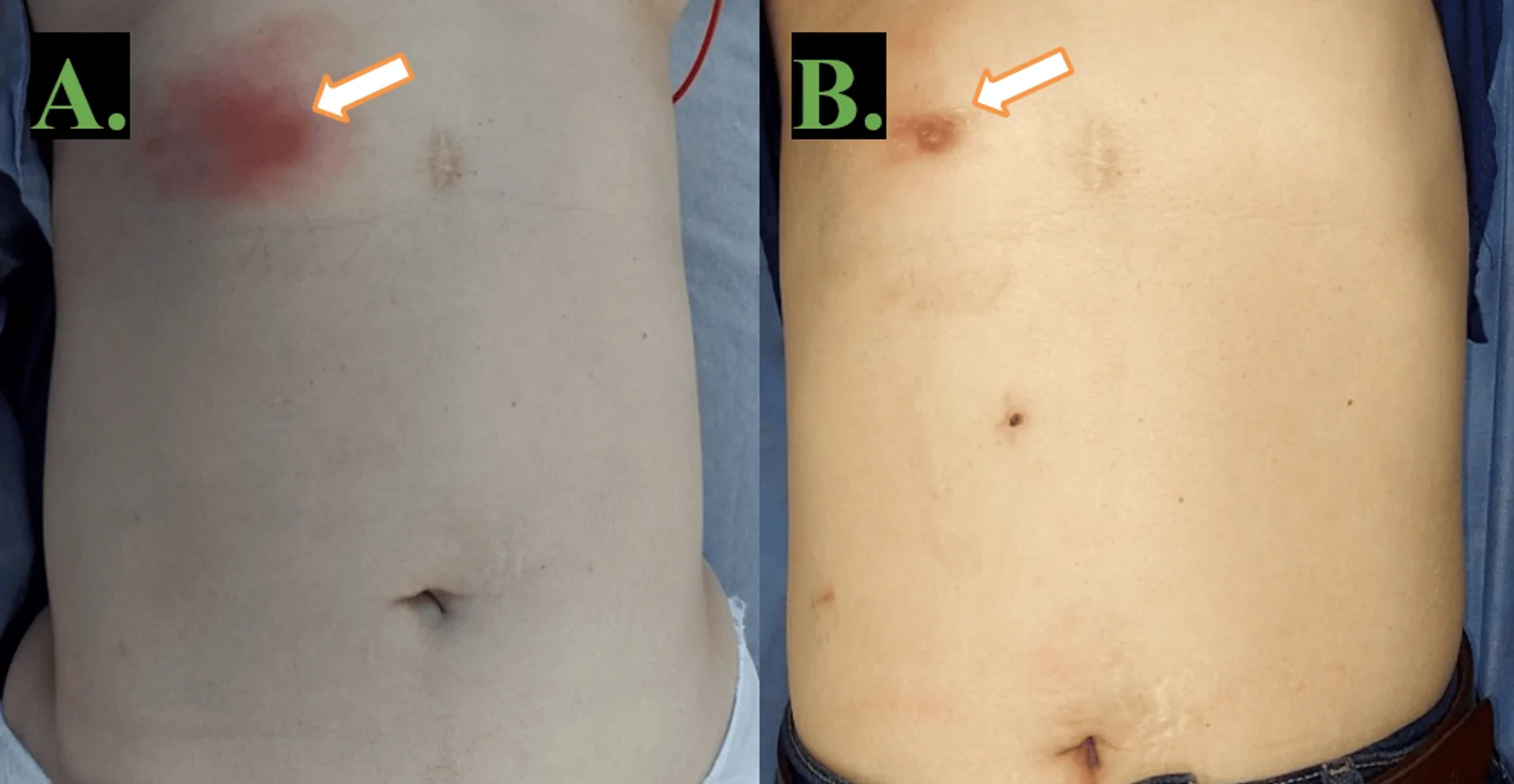
Comparison before and after surgical management (A) Swelling of the right costal margin, measuring approximately 8 cm in diameter, with overlying erythema (white arrow). (B) One-month postoperative follow-up demonstrating marked reduction in erythema and soft-tissue swelling.

## Discussion

LC is the surgical treatment of choice for symptomatic cholelithiasis and acute cholecystitis [8-10]. However, gallbladder perforation with spillage of bile and gallstones into the peritoneal cavity remains a recognized complication [9]. Although most spilled gallstones remain clinically silent, a small proportion of patients may develop long-term complications [2]. Among these, abscess formation is the most frequently reported complication, accounting for up to 60% of symptomatic cases [2].

The diagnosis of complications related to retained gallstones can be challenging because of their heterogeneous clinical presentation, delayed onset, variable imaging findings, and the frequent absence of documentation of gallbladder perforation or stone spillage in the original operative report [10]. These factors may lead to diagnostic uncertainty and may initially raise concern for neoplastic disease, particularly when a chronic inflammatory mass develops in an unusual location. In the present case, the combination of a slowly progressive subcostal mass and a chronic inflammatory collection initially prompted evaluation for a chest wall neoplasm. Retrospective review of the MRI, however, revealed a small hypointense nodular structure within the collection, which corresponded to the retained gallstone identified during surgery.

Prevention remains the most important strategy for minimizing complications related to spilled gallstones. Several measures have been proposed to reduce the risk of stone spillage, including decompression of the gallbladder before extraction, enlargement of the fascial incision when necessary, and use of retrieval bags for specimen removal [11]. When gallbladder perforation occurs, efforts should be made to retrieve all visible stones and irrigate the peritoneal cavity with saline. The use of a retrieval bag is recommended even when the gallbladder has been perforated during extraction [11]. Routine conversion to open surgery is not recommended solely because of gallstone spillage [11].

Management of complications from retained gallstones depends largely on their location and clinical presentation. Abdominal wall abscesses may be managed with local drainage, whereas intra-abdominal collections may require percutaneous drainage. Laparoscopic or open surgical exploration may be necessary when percutaneous drainage is unsuccessful or when definitive removal of the retained stone is required [11].

This case highlights the importance of considering retained gallstones in the differential diagnosis of delayed abdominal or chest wall inflammatory masses in patients with a history of LC, even when gallbladder perforation or stone spillage was not documented during the original procedure. Careful assessment of imaging findings, particularly the identification of a foreign-body-like structure within a chronic collection, may provide an important diagnostic clue and help avoid an unnecessary or delayed oncologic workup.

## Conclusions

Retained gallstones following LC are an uncommon but potentially significant cause of delayed intra-abdominal and abdominal wall complications. Their diagnosis can be challenging because symptoms may develop years after surgery and may mimic other inflammatory or neoplastic conditions. A history of LC should therefore be specifically sought in patients presenting with unexplained chronic abdominal or abdominal wall collections. When retained gallstones are suspected, detailed review of prior operative records and imaging, combined with a high index of clinical suspicion, may help establish the diagnosis and prevent unnecessary investigations for malignancy. Finally, prevention remains fundamental. Every effort should be made to avoid gallbladder perforation and to retrieve all spilled gallstones when perforation occurs. When stones cannot be completely retrieved, accurate documentation of gallbladder perforation and stone spillage in the operative report is essential to facilitate recognition of potential delayed complications.
